# Corrigendum: A Music-Therapy Robotic Platform for Children With Autism: A Pilot Study

**DOI:** 10.3389/frobt.2022.965369

**Published:** 2022-07-08

**Authors:** Huanghao Feng, Mohammad H. Mahoor, Francesca Dino

**Affiliations:** ^1^ Changshu Institute of Technology, Suzhou, China; ^2^ Computer Vision and Social Robotics Labarotory, Department of Electrical and Computer Engineering, University of Denver, Denver, CO, United States

**Keywords:** social robotics, autism, music therapy, turn-taking, motor control, emotion classification

Missing information in Acknowledgment Section

In the published article, there was an error regarding the **acknowledgment** section. Additional information needs to be added to this section. Rephrased acknowledgment as follows: “This research was conducted at the University of Denver (DU) as HF’s Ph.D. dissertation. The research was partially supported by the Autism gifts given to the DU by several family members. We would like to thank the families who donated to DU in support of this research as well as those families and their children who participated in this research. The publication cost was covered by the University of Denver (50%) and MOE Project of Humanities and Social Sciences (21YJCZH029) (50%).”

The authors apologize for this error and state that this does not change the scientific conclusions of the article in any way. The original article has been updated.

